# Synthesis and Corrosion Inhibition Potential of Cerium/Tetraethylenepentamine Dithiocarbamate Complex on AA2024-T3 in 3.5% NaCl

**DOI:** 10.3390/ma15196631

**Published:** 2022-09-24

**Authors:** Thi Huong Pham, Woo-Hyuk Lee, Gyeong-Ho Son, Trang Thu Tran, Jung-Gu Kim

**Affiliations:** 1 School of Advanced Materials Science and Engineering, Sungkyunkwan University, 2066, Seobu-Ro, Jangan-Gu, Suwon 440-746, Korea; 2 Department of Energy Science, Sungkyunkwan University, 2066, Seobu-Ro, Jangan-Gu, Suwon 440-746, Korea

**Keywords:** aluminium, alloy, corrosion inhibitor, synthesized inhibitor, XPS, passive films

## Abstract

In this work, a cerium/tetraethylenepentamine dithiocarbamate complex was synthesized and evaluated for the corrosion inhibition capability on an AA2024-T3 Al alloy in a 3.5% NaCl medium. The synthesized compounds were characterized via spectroscopic techniques. The corrosion inhibition behaviour of the complex was elucidated by electrochemical measurements and surface analysis techniques. Based on electrochemical test results, the corrosion inhibition efficiency of the complex increases with the immersion time of aluminium alloy in the test solution. The corrosion inhibition reaches 96.80% when the aluminium is immersed in a 3.5% NaCl solution containing a corrosion inhibitor for 120 h. The potentiodynamic polarization test results show that the complex acts as a mixed-type corrosion inhibitor and the passive range is widened. The surface analysis methods reveal that the corrosion inhibition ability of the complex originated from the formation of a protective layer on the Al surface. This film is created from the physisorption and chemisorption of cerium ions and organic parts simultaneously released from the complex molecules.

## 1. Introduction

The AA2024-T3 aluminium alloy is widely applied in transportation fields such as aerospace due to its high strength-to-weight ratio and resistance to damage. These significant advantages come from the substantial presence of copper (Cu) and magnesium (Mg) elements in the texture of the alloy. However, the existence of these elements can also cause the localized corrosion susceptibility of AA2024-T3 in chloride environments containing oxygen or an oxidant. During the alloying process, these elements exist within main intermetallic particles (IMPs) including Al_2_CuMg (S phase), Al_2_Cu (Θ phase), and Al_7_Cu_2_Fe [1]. According to Buchheit et al., in the components of IMPs, S phases with particles around 500 nm in diameter are predominant and make up 61.3%, the second most abundant type is the Al_6_(Cu, Mn, Fe) phase with 12.3%, the Al_7_Cu_2_Fe phase makes up 5.2%, followed by the (Al, Cu)_6_ phase with 4.3%. In the ambient atmosphere, an oxide layer with a thickness of a few nanometres is formed on the AA2024-T3 surface and protects it from corrosion. However, when this alloy is exposed to moisture or/and electrolyte, the oxide layer is inadequate to defend it from corrosion because of the formation of a microgalvanic coupling between IMPs and the Al metallic matrixes [2]. According to various studies about corrosion behaviour of AA2024-T3 in chloride environments, the S phases are active phases and Θ phases play a role as noble phases. At the beginning of the corrosion process, with a copious presence on the AA 2024-T3 alloy surface, the S phases undergo a de-alloying process with the chemical and electrochemical dissolution of Al and Mg of the S phases, leading to the Cu-rich S phases. Consequently, the de-alloyed S phases are cathodic sites while the Al metallic matrixes are anodic sites, and are corrodent preferentially. Therefore, the corrosion of AA2024-T3 is suppressed when the de-alloying behaviour of S phases is reduced, and the Al metallic matrix is protected from localized corrosion [1,2,3,4,5].

Various methods have been applied to enhance the corrosion resistance of this alloy in NaCl solution including coatings and using corrosion inhibitors [6]. In particular, the use of corrosion inhibitors is a major focus due to their low utilization cost and potent protection efficiency [7]. The corrosion inhibitors for AA2024-T3 in NaCl solution include *ocimum basilicium seeds extract* [8], *thiosemicarbazone derivatives* [1], *cerium (III) and cerium (IV) salts* [9], *copper complexing compounds* [10], *sodium diethyldithiocarbamate* [11], *sodium silicate* [12], and *chromate compounds* [13,14,15,16]. Of the reported corrosion inhibitors, chromate compounds have shown great corrosion inhibition abilities, but yield an adverse impact on the environment because of their high toxicity [17]. This has introduced challenges for scientists in terms of finding anti-corrosion compounds that are not only effective but also safe for the environment.

Owing to their eco-friendly properties, rare-earth metal compounds (lanthanide (La) and cerium (Ce)) have been studied extensively as a substitute for chromate compounds as corrosion inhibitors for metals [18,19,20]. In particular, cerium salts (CeCl_3_, Ce(NO_3_)_3_) have exhibited outstanding effectiveness in the corrosion protection for Al alloys [21], originating from the formation of Ce(OH)_3_ precipitates, which are then oxidized to Ce(OH)_4_. Ce^3+^ and Ce^4+^ compounds deposit on cathodic sites and inhibit oxygen reduction reactions [9]. Moreover, Ce ion-loaded organic compounds synthesized from Ce inorganic salts and organic compounds have been developed [22]. This method has decreased the number of organic compounds used and shows marvellous corrosion inhibition which is the result of the synergistic performance of both Ce ions and organic constituents. The anodic sites of metals are protected through the adsorption of organic parts while Ce ions adsorb and conserve the cathodic sites of metals [23]. Moreover, organic parts in the form of large structures containing heteroatoms (N, O, P, and S) produce an advanced corrosion inhibition ability due to their large structures generating an immense covered metal surface screen while N, O, P, and S atoms act as adsorption centres.

With regard to the organic compounds used for synthesizing Ce ion-loaded organic corrosion inhibitors, dithiocarbamate compounds containing dithiocarbamato (N−CS_2_^−^) functional groups have been applied as effective corrosion inhibitors for Al alloys [11,24]. This is because N−. CS_2_^−^ groups in these compounds interact with the Al alloy surface through the electrons shared between S atoms and Al, leading to the formation of a protective film on the Al surface. Meanwhile, metal–dithiocarbamate complexes deposit directly on the Al surface, protecting it from the strong attack of a corrosive environment [22]. Recently, there have been many studies regarding the use of a combination of Ce salts and dithiocarbamate compounds as corrosion inhibitors for AA2024-T3 in a 3.5% NaCl solution. Two compounds are either applied simultaneously into corrosive media [11,24] or are combined to form a new complex, and used as a corrosion inhibitor [22]. Moreover, almost all investigated corrosion inhibitors for AA2024-T3 in NaCl solution in two cases originated from diethyldithiocarbamate compounds (4 C—a short alkyl chain). There have not been any studies on long alkyl chain dithiocarbamate corrosion inhibitors. Long-chain dithiocarbamate corrosion inhibitors show effective corrosion inhibition on the Al alloys not only due to the interaction between N-CS_2_^−^ groups and Al surface but also due to the high metal surface screen effect of large compounds [25]. In particular, the larger the number of NH functional groups, the larger the number of replacement positions for CS_2_^−^ groups. This leads to the excellent binding capacity of N−CS_2_^−^ with metallic ions [26].

This study is the first time that a dithiocarbamate with a long alkyl chain structure (8 C) has been synthesized, combined with a metallic salt, and tested as a corrosion inhibitor for AA 2024-T3 alloy in a 3.5% NaCl corrosive environment. This electrolyte was chosen based on ASTM G44 specification, which is standard practice for metals and alloys susceptible to chloride ions. Moreover, a 3.5% NaCl solution was selected to approach the conditions of seawater, which has high humidity [8]. The synthesized compounds were characterized using Fourier transform-infrared spectroscopy (FT-IR) and X-ray diffraction spectroscopy (XRD) techniques. The corrosion inhibition effectiveness was determined via electrochemical methods. Moreover, the inhibition mechanism was revealed via scanning electron microscopy-energy dispersive spectroscopy (SEM-EDS) and X-ray photoelectron spectroscopy (XPS).

## 2. Materials and Methods

### 2.1. Materials

Tetraethylenepentamine (TEPA, technical grade), cerium (III) chloride heptahydrate (CeCl_3_. 7H_2_O, 98%), and carbon disulphide (CS_2_, 99.9%) were purchased from Sigma-Aldrich Co., Ltd., Seoul, Korea. Sodium chloride (NaCl, 99.5%) and sodium hydroxide (NaOH, 98%) were provided by Samchun Chemical Co., Ltd., Seoul, Korea. Ethanol (EtOH), acetone, acetonitrile, and AA 2024-T3 alloy were purchased from local Korean companies. The chemical composition of the AA 2024-T3 alloy used was 0.45% Si, 0.45% Fe, 0.46% Mn, 1.2–1.8% Mg, and 3.8–4.9% Cu, with the remaining balance being Al.

### 2.2. Synthesis Procedures

#### 2.2.1. Preparation of the Dithiocarbamate Ligand—Sodium Tetraethylenepentamine Dithiocarbamate (TEPA/CSSNa)

**Figure 1:**
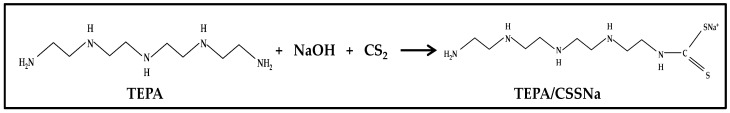
Scheme of the synthesis of the dithiocarbamate ligand-TEPA/CSSNa.

TEPA/CSSNa was synthesized following a recent study with a slight modification; the reaction can be seen in Figure 1 [27]. First, TEPA (0.76 g, 4 mmol) and NaOH (0.16 g, 4 mmol) in 20 mL of acetonitrile were stirred for 20 min in a cold mixture of ice at 0–5 °C. After that, CS_2_ (0.3 g, 4 mmol) was added drop-wise into the mixture and stirred continuously for 3 h in an ice bath and for 1 h at room temperature to form a light-yellow solution. Next, the solvent was removed via rotary evaporation, yielding a light yellow solid product. This solid was rinsed with EtOH to remove any unreacted amine and was dried under vacuum. The light-yellow solid powder was characterized via FT-IR.

#### 2.2.2. Synthesis of the Metal–Dithiocarbamate Coordinate Compound—Cerium/Ditetraethylenepentamine Dithiocarbamate ((TEPA/CSS)_2_Ce) Complex

The synthesis process of the (TEPA/CSS)_2_Ce complex was based on a common synthetic procedure of metal–dithiocarbamate coordinate compounds by the addition of metallic salts to dithiocarbamate ligands [28]. The (TEPA/CSS)_2_Ce complex was synthesized using the above synthesized TEPA/CSSNa dithiocarbamate ligand and CeCl_3_ 7H_2_O salt. First, CeCl_3_ 7H_2_O salt (0.37 g, 1 mmol) was dissolved in 10 mL of distilled water. Then, this CeCl_3_ solution was slowly added to the solution of TEPA/CSSNa (0.58 g, 2 mmol) in 15 mL of acetonitrile; the mixture was stirred at room temperature for 30 min. The colour of the solution immediately changed due to the formation of a precipitate while the CeCl_3_ solution was added. This precipitate was filtered and rinsed with EtOH to yield a wet crude product. This product was dried in an oven at 50 °C to obtain a light brown solid [27]. This solid was characterized via FT-IR and XRD techniques and was used as a corrosion inhibitor for AA 2024-T3 alloy in a 3.5% NaCl solution.

### 2.3. Characterization of the Synthesized Compounds

The formation of TEPA/CSSNa was confirmed using FT-IR (Nicolet iS50, wave numbers region from 4000 cm^−1^ to 400 cm^−1^). The FT-IR technique was also used to confirm the formation of the synthesized (TEPA/CSS)_2_Ce compounds. The crystal phases of (TEPA/CSS)_2_Ce were studied using the high-resolution X-ray diffraction (HR-XRD) technique. XRD pattern was recorded with an XRD diffractometer using Cu, K_α_, and diffraction angles of 0° and 100° (Rigaku Corporation, Tokyo, Japan).

### 2.4. Corrosion Media

3.5% NaCl solution was prepared from the dissolution of 99.5% NaCl into distilled water; this solution was used as the corrosion environment. A total of 1 g of synthesized (TEPA/CSS)_2_Ce complex was added into 1 L of 3.5% NaCl solution followed by stirring for 24 h. After that, this mixture was filtered to separate the filtrate and residue. While the filtrate was utilized as a test solution, the residue was dried and weighed to calculate the concentration of the ((TEPA/CSS)_2_Ce) corrosion inhibitor in the test solution. The synthesized complex inhibitor was used in tests at a concentration of 500 ppm.

### 2.5. Electrochemical Tests

AA2024-T3 plates were cut into test samples with sizes of 3 cm × 2 cm × 0.2 cm. They were then polished using 400, 600, and 1200 grade SiC emery paper. Next, the samples were ultra-sonicated in acetone for 10 min and rinsed using EtOH and distilled water prior to each test.

Electrochemical measurements including electrochemical impedance spectroscopy (EIS) and potentiodynamic polarization were performed using a VSP-300 (Biologic Science Instruments, Seyssinet-Pariset, France) potentiostat with a three-electrode system. This system consisted of a working electrode (AA2024-T3 samples with 1 cm^2^ of contact area), a reference electrode (saturated calomel electrode (SCE)), and a graphite counter electrode. The electrodes were washed with distilled water prior to each experiment.

EIS tests were carried out in the frequency range from 100 kHz to 10 mHz with a 10 mV amplitude at open-circuit potential (OCP). The corrosion inhibition efficiency (*E*) was calculated from the following equation [29,30]:

(1) $$E=\frac{R_{p,p}-R_{p,a}}{R_{p,p}}\times 100\%$$

where $R_{p,p}$ and $R_{p,a}$ represent the polarization resistances in the presence and absence of a corrosion inhibitor, respectively.

Potentiodynamic polarization tests were performed with a sweep rate of 0.166 mV s^−1^. Tafel curves were obtained in the potential range from −15 V (vs. SCE) to 0 V (vs. SCE) around OCP. The parameters were collected from Tafel curves containing the corrosion potential (*E_corr_*) and corrosion current density (*I_corr_*). The corrosion inhibition efficiency (*E*) was calculated according to the following equation [31,32]:

(2) $$E=\frac{I_{corr,\;a}-I_{corr,\;p}}{I_{corr,\;a}}\times 100\%$$

where $I_{corr,\;a}$ and $I_{corr,\;p}$ are the corrosion current densities without and with corrosion inhibitor, respectively.

### 2.6. Surface Characterization

SEM-EDS (JSM-7900F) was applied to examine the change in surface morphology as well as the elemental composition presented on the alloy surface after alloy samples were immersed in NaCl solution in the absence and presence of the inhibitor [33,34]. Furthermore, FT-IR (Nicolet iS50, wave numbers region from 4000 cm^−1^ to 400 cm^−1^) and XPS (ESCALAB250) techniques were used to study the chemical composition and formation mechanism of the product layer on the surface of these alloy samples [35].

## 3. Results

### 3.1. Characterization of the Synthesized Compounds

#### 3.1.1. FT-IR

##### TEPA/CSSNa

**Figure 2:**
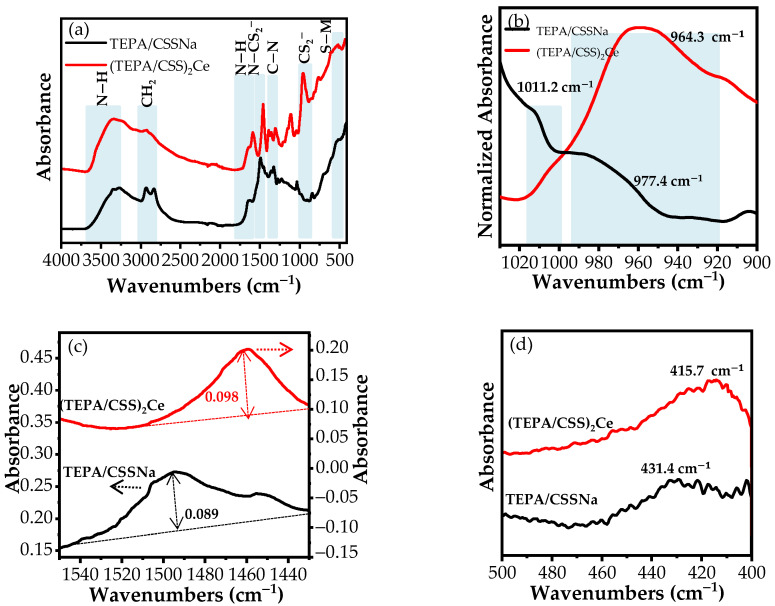
FT-IR spectra of (**a**) synthesized TEPA/CSSNa and (TEPA/CSS)_2_Ce compounds, (**b**) CS_2_^−^ groups, (**c**) N−C in N− CS_2_^−^ groups, and (**d**) S− M bonds (M: metals).

The FT-IR technique was applied to investigate the functional groups of synthesized compounds TEPA/CSSNa and (TEPA/CSS)_2_Ce, as shown in Figure 2a. In the spectrum of TEPA/CSSNa, a broad peak split into two small peaks located at 3354.6 and 3275.7 cm*^−^*^1^ indicates the N−H groups in amines, and the peak at 1635.1 cm*^−^*^1^ is due to the N−H bends in amines. Two peaks at 2934.1 and 2830.7 cm*^−^*^1^ are attributed to the CH_2_ groups. The double peaks at 1493.8 and 1452.8 cm*^−^*^1^ correspond to N−C bonds in N−CS_2_^−^ groups. The two peaks located at 1362.5 and 1329.6 cm*^−^*^1^ signify the C−N stretching of amines. The C−S bonds are observed at 1011.2 and 977.4 cm*^−^*^1^, and the bands at 431.4 cm*^−^*^1^ are linked to the existence of S-Na bonds (Figure 2a).

##### (TEPA/CSS)_2_Ce

The peaks observed at 3357.1 and 3235.8 cm^−1^ represent the existence of the N−H groups in the synthesized compound. The adsorption bands observed at 2922.5 and 2858.5 cm^−1^ are assigned to CH_2_ groups. A sharp peak at 1649.9 cm^−1^ is related to the presence of NH bends in amines. The peak at 1461.3 cm^−1^ is ascribed to N−C bonds in N−. CS_2_^−^ groups, and the peaks at 1389.1 and 1362.9 cm^−1^ represent the C−N stretching of amines. A peak detected at 964.3 cm^−1^ is due to the presence of C−S bonds and the peak at 415.7 cm^−1^ is attributed to S−Ce bonds (Figure 2a).

Dithiocarbamate ligands coordinate with metal ions to generate complexes with different structures formed by either bidentate symmetrical bonding or monodentate unsymmetrical bonding [36]. According to A. Jayaraju et al., the number of peaks indicated that C−S bonds are closely related to the bonding mode between N−CS_2_^−^ groups and metal ions. Therefore, the FT-IR spectra in the range of 1030 cm^−1^ and 900 cm^−1^, where C−. S bonds are shown for TEPA/CSSNa and (TEPA/CSS)_2_Ce, are exhibited in Figure 2b. As shown in Figure 2b, the FT-IR spectrum of TEPA/CSSNa shows two peaks located at 1011.2 cm^−1^ and 977.4 cm^−1^, while only one peak positioned at 964.3 cm^−1^ is observed on the FT-IR spectrum of (TEPA/CSS)_2_Ce in this wavenumber range. The appearance of two peaks in the case of TEPA/CSSNa expresses the monodentate coordination between CS_2_^−^ groups and Na ions. In the case of (TEPA/CSS)_2_Ce, the single peak is an indication of bidentate coordination meaning that both S atoms of CS_2_^−^ groups participate in the coordination with Ce ions [37].

Furthermore, the change in absorbance value of the peaks representing N−C bonds in N−CS_2_^−^ groups is also studied to confirm the formation of the complex. According to studies about the formation mechanism of metal–dithiocarbamate compounds, the net effect of ӆ-electron flow from N atoms to S atoms in N−CS_2_^−^ groups is a ligand that has forceful electron σ-donor properties, leading to the transfer behaviour of electron density from S atoms to the metal centres, favouring the stability of compounds. Moreover, the strong σ-donor properties of dithiocarbamate ions are conducive to the formation of compounds having high oxidation states (Ce ions) instead of low oxidation states (Na ions) [38]. The absorbance value of the N−C peak in the case of (TEPA/CSS)_2_Ce is 0.098, which is higher than that of TEPA/CSSNa with 0.089 (Figure 2c). This indicates that the electron density transfer of S atoms to the metal centre in the formation process of (TEPA/CSS)_2_Ce is greater than that of TEPA/CSSNa. This is consistent with the bidentate coordination state of (TEPA/CSS)_2_Ce compared to the monodentate coordination of TEPA/CSSNa. The coordination between the CS_2_^−^ groups and Ce ions result in the formation of the (TEPA/CSS)_2_Ce complex. The negative shift in the peak position of the C−S bond in the (TEPA/CSS)_2_Ce compound compared to the TEPA/CSSNa compound also confirms the formation of the (TEPA/CSS)_2_Ce complex (Figure 2b) [39].

Furthermore, on the FT-IR spectra of TEPA/CSSNa and (TEPA/CSS)_2_Ce, there is an appearance of peaks at 431.4 cm^−1^ and 415.7 cm^−1^, respectively (Figure 2d). These peaks correspond to new bonds formed between S atoms and metal ions [40]. FT-IR results confirm the high presence probability of dithiocarbamate ligands and metal–dithiocarbamate coordinating compounds in the synthetic products.

#### 3.1.2. XRD

**Figure 3:**
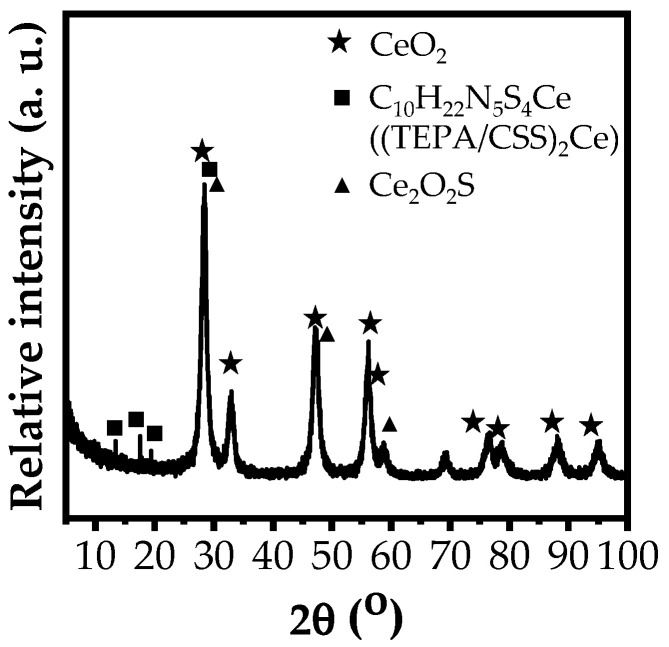
XRD spectrum of the synthesized (TEPA/CSS)_2_Ce complex.

The XRD spectrum and crystalline phases of the (TEPA/CSS)_2_Ce compound are shown in Figure 3.

The XRD pattern in Figure 3 shows diffraction peaks at 2Θ° values of 88.40, 78.88, 76.42, 69.15, 56.18, 57.52, 47.12, 32.78, and 28.32° corresponding to the existence of the CeO_2_ phase [41,42]. The diffraction peaks at 58.86, 47.66, and 28.64° are assigned to the Ce_2_O_2_S phase [43]. The other peaks at 13.42, 17.44, 19.36, 25.62, and 28.32° are related to the presence of complexes formed from the coordination between metal ions and isolated electron pairs of S atoms in dithiocarbamates [44]. In this case, they are evidence of the existence of the C_10_H_22_N_5_S_4_Ce ((TEPA/CSS)_2_Ce) complex.

**Figure 4:**
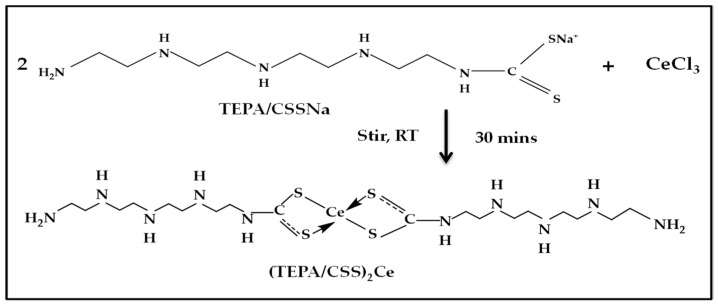
The synthesis and molecular structure of (TEPA/CSS)_2_Ce.

From the obtained characterization results, TEPA/CSSNa and (TEPA/CSS)_2_Ce compounds were successfully synthesized. The molecular structure, as well as the synthetic reaction of (TEPA/CSS)_2_Ce, is shown in Figure 4.

### 3.2. Electrochemical Studies

#### 3.2.1. EIS Measurements

**Figure 5:**
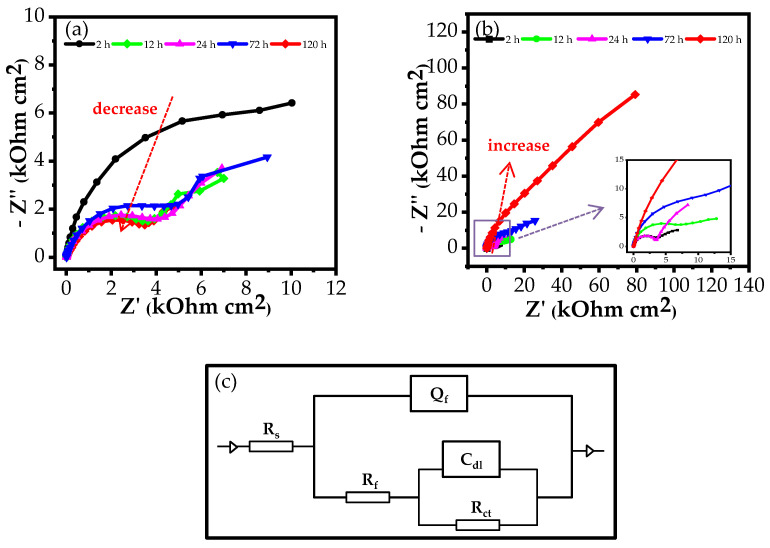
Nyquist plots of AA2024-T3 in (**a**) 3.5% NaCl solution, (**b**) 3.5% NaCl solution in the presence of (TEPA/CSS)_2_Ce with immersion time, and (**c**) the corresponding equivalent circuit for EIS curves of the Al alloy in 3.5% NaCl and 3.5% NaCl-containing (TEPA/CSS)_2_Ce.

The Nyquist plots of AA2024-T3 in 3.5% NaCl in the absence and presence of (TEPA/CSS)_2_Ce complex for 120 h are shown in Figure 5.

As observed from Figure 5a,b, the Nyquist plots in the two cases show the same shapes containing two capacitive loops with a large capacitive loop located in the high-frequency range and a small capacitive loop located in the low-frequency range. This means that there is no change in the corrosion reaction mechanism of the Al alloy in NaCl either due to an increase in immersion time or the presence of a corrosion inhibitor [45,46]. The large capacitive loops are related to the creation of Al^3+^, OH^−^, and O^2-^ ions in the charge transfer process at the metal/oxide/electrolyte interface while the small capacitive loops are generated by the diffusion of ions through the corrosion product layer or inhibitor film [1].

An equivalent circuit was fitted to the EIS curves using Zsimpwin software to elucidate the electrochemical behaviour and determine the specific electrochemical parameters [47]. This circuit is shown in Figure 5c, and the corresponding parameters are listed in Table 1. The equivalent circuit is the combination of elements including the solution resistance (R_s_), the resistance of the surface film (R_f_), the capacitance of the surface film (Q_f_), double-layer capacitance (C_dl_), and charge transfer resistance (R_ct_).

Because the Al alloy surface is rough and heterogenous, the Q_f_ element is used in lieu of an ideal capacitance [48]. The impedance of Q_f_ is defined using the following equation [49,50]:

(3) $$Z_{Q}=Y_{0}{}^{-1}\times {\left(jw\right)}^{-n}$$

where $Y_{0}$ denotes the constant of Q, *j* is the imaginary number, *w* expresses the angular frequency, and n represents the phase shift (−1 < *n* < 1). The roughness of the metal surface can be assessed using the n value, where a small n value represents a rough surface [8].

According to Equation (1), the inhibition efficiency (*E*) values were calculated through the polarization resistance (*R_p_*) values. Here, *R_p_* values were extracted through the surface film resistance (*R_f_*) and charge transfer resistance (*R_ct_*) values using the following equation [8]:

(4) $$R_{p}=R_{f}+R_{ct}$$

**Figure 6:**
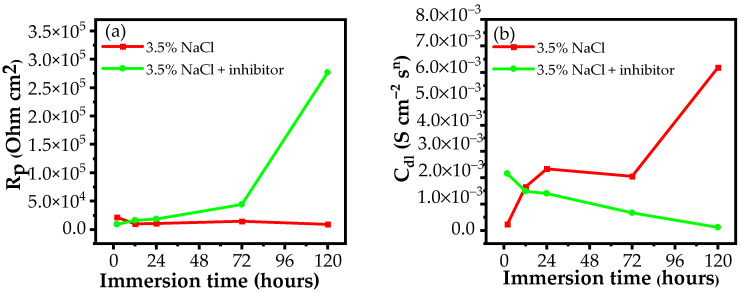
(**a**) The *R_p_* values, and (**b**) the *C_dl_* values of AA2024-T3 in 3.5% NaCl in the absence and presence of a corrosion inhibitor versus immersion time derived from the EIS curves.

As can be seen in Table 1 and Figure 6a, at the same embedding time, the *R_p_* value for the Al sample immersed in a 3.5% NaCl-containing inhibitor is larger than in 3.5% NaCl except at 2 h. This expresses that the complex exhibits the corrosion inhibition ability for the Al alloy in all immersion times except 2 h [51].

When the Al alloy is immersed in 3.5% NaCl corrodent, the *R_p_* value decreases during the initial 12 h followed by a slight increase over the next 12 h. This originated from the local corrosion of Al in a corrosive NaCl environment. After that, the R_p_ value gradually increases over time to reach 14.58 ×10^3^ at 72 h due to the formation of an Al_2_O_3_ oxide layer on the Al surface [24]. The following is a significant drop-off of *R_p_* value when the Al_2_O_3_ layer is attacked and penetrated by aggressive Cl^−^ ions [52].

While the *R_p_* value of the Al alloy in the blank solution declined, the *R_p_* value of Al in the NaCl-containing inhibitor is boosted with immersion time. This behaviour is related to the creation of a protective layer on the Al surface by the absorption of the inhibitor molecules [53,54]. When the immersion time is prolonged, more inhibitor molecules are adsorbed onto the Al alloy surface, leading to an increase in the surface coverage. As a result, the protection effectiveness increases and the *R_p_* value increases [55]. The higher the *R_p_* value, the better the corrosion inhibition (*E*) efficiency. The *E* value is 33.55% during the first 12 h of immersion time; this value reaches 96.80% when the immersion time is extended to 120 h.

The relationship between the *C_dl_* value and the thickness of the adsorbed layer on the Al surface is given by the following equation [56,57]:

(5) δads=εε0ACdl

where $\delta_{ads}$ is the thickness of the adsorbed layer on the metal surfaces, ε represents the permittivity of air, $\varepsilon_{0}$ expresses the constant of local dielectric, and A is the surface area of the metal electrodes.

As clearly seen in Figure 6b, the *C_dl_* value of the Al alloy sample in the inhibitor-free solution rises during the survey period. By contrast, a downtrend in the *C_dl_* value from 2.16 × 10^−3^ to 0.12 × 10^−3^ S cm^−2^s^n^ is observed when the corrosion inhibitor is added to a 3.5% NaCl solution. The reduction in the *C_dl_* value reflects an enhancement in the value of $\delta_{ads}$, indicating a thicker adsorbed layer on the AA2024-T3 alloy over time [58].

#### 3.2.2. Potentiodynamic Polarization Measurements

**Figure 7:**
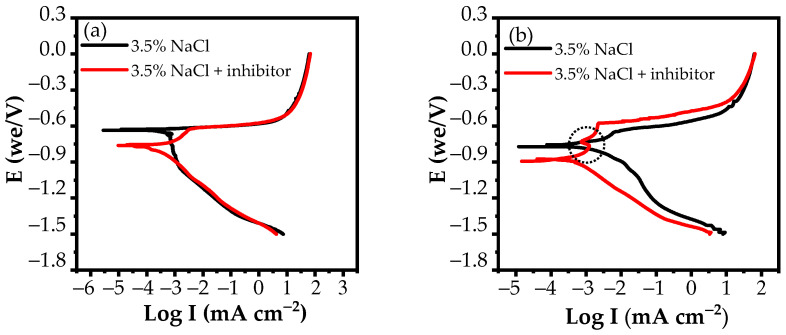
Potentiodynamic polarization curves of AA 2024-T3 in a 3.5% NaCl-containing inhibitor (**a**) at 24 h of immersion time and (**b**) at 120 h of immersion time.

Figure 7 shows the potentiodynamic polarization curves obtained on AA 2024-T3 alloy in a 3.5% NaCl solution in the absence and presence of an inhibitor at two immersion times. Electrochemical parameters including the corrosion potential (*E_corr_*), corrosion current density (*I_corr_*), and inhibition efficiency (*E*) are extracted and presented in Table 2.

Figure 7 illustrates that the corrosion potential (*E_corr_*) values of the Al alloy immersed for both 24 h and 120 h are shifted towards the negative direction when the corrosion inhibitor is presented in a 3.5% NaCl solution compared to the blank solution. The differences in the *E_corr_* values are about 140 mV and 130 mV at immersion times of 24 h and 120 h, respectively. This is indicative of cathodic-type corrosion inhibitors [59]. However, after 24 h of exposure time, a small passive region (*E_pi_*_t_−*E_corr_*) is formed in the range of 0.15 V when the corrosion inhibitor is presented in a 3.5% NaCl solution, although the passive region is not observed in the case of the blank solution. This behaviour is also inspected when the immersion time is extended to 120 h. After 120 h of immersion time, the passive range is enlarged by 0.22 V in the presence of an inhibitor in NaCl solution. Moreover, the current densities of both the anodic and cathodic branches are reduced substantially as the Al alloy is immersed in NaCl solution with corrosion inhibitor for 120 h. The above results prove that the inhibitor acts as a mixed-type corrosion inhibitor with the domination of cathodic reaction inhibition. This means that the inhibitor not only retards the cathodic reactions but also the anodic reactions.

Table 2 shows that there is a decline in the (*I_corr_*) corrosion current density values with the immersion time when a corrosion inhibitor is added to the NaCl solution. Longer immersion time results in more obvious reductions of the *I_corr_* value. After 24 h of exposure time, the *I_corr_* value of Al alloy in 3.5% NaCl-containing inhibitor is decreased by 158 μA cm^−2^ compared to the blank solution. When the immersion time is extended to 120 h, this decrease is 1953 μA cm^−2^. As a result, the corrosion inhibition efficiency (*E*) of the complex on AA 2024-T3 in 3.5% NaCl increases with time. The *E* value is 32.31% when the immersion time is 24 h; this value reaches 81.72% when the immersion time is 120 h. These results are consistent with the results obtained from EIS measurements.

It is worthwhile to note from Figure 7b that a flicker occurs on the anodic branch after the Al alloy is immersed in a 3.5% NaCl-containing inhibitor for 120 h. This is presumed to be due to the electrochemical transformation of the surface film [60]. This behaviour is due to the transition from the IMPs’ active areas to the passive areas which are adsorbed by the inhibitor molecules.

### 3.3. Surface Characterization

#### 3.3.1. SEM-EDS Analysis

**Figure 8:**
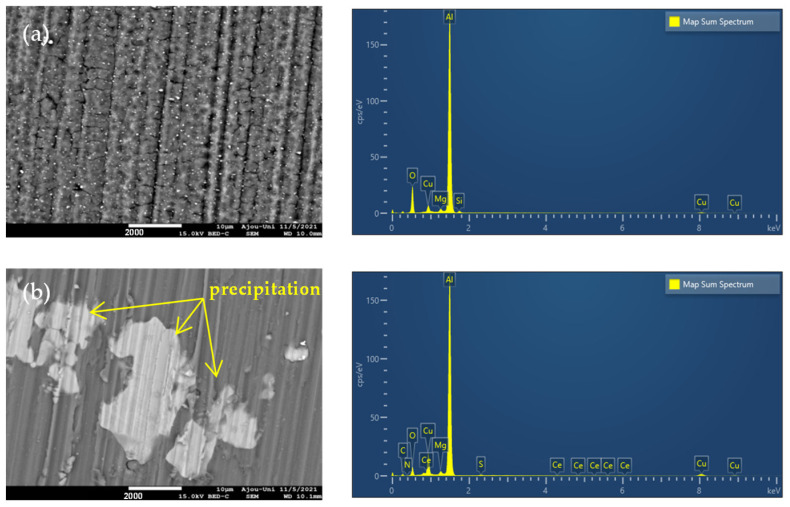
SEM images and EDS spectra of AA 2024-T3 alloy in (**a**) 3.5% NaCl and (**b**) 3.5% NaCl-containing inhibitor for 120 h.

Figure 8 shows SEM images as well as the EDS spectra of AA2024-T3 alloy samples immersed in a 3.5% NaCl solution in the absence and presence of a corrosion inhibitor for 120 h. Furthermore, the elemental composition corresponding to the product layer formed on the Al alloy surface is listed in Table 3.

As shown in Figure 8a, after the AA2024-T3 alloy is immersed in 3.5% NaCl for 120 h, the sample is rough with many corrosion pits. This is a consequence of the strong attack of aggressive (Cl^−^) anion in the corrosive medium on the AA 2024-T3 alloy surface [61]. The pitting corrosion behaviour of the AA 2024-T3 alloy is related to the dissolution of the second phase particles, expressly (Al_2_CuMg) S phase particles. When the Al alloy is immersed in the NaCl solution, a microgalvanic cell is created between the S phase and AA2024-T3 matrix because of the potential difference of 300–400 mV. Because the S phase has a more negative potential, the S phase plays a role as an anode and the AA2024-T3 matrix acts as a cathode. Consequently, the S phase is more active and is abruptly dissolved [62]. Since Mg is dissolved preferentially from the S phase particles, the S phase undergoes a de-alloying behaviour. As a result, the S-phase remnants are mostly Cu-rich particles, leading to the subsequent cathodic behaviour [63]. This is proven by the presence of Cu with 5.3%, which is significantly larger than Mg (0.9%) in the composition of the surface product layer (Figure 8a, Table 3). In parallel, the Al metal matrix is also oxidized by means of uniform corrosion, leading to the formation of an Al_2_O_3_ layer on the metal surface [64]. Additionally, this oxide layer is the reason for the abundant presence of oxygen (21.6%) in the composition of the surface layer.

In comparison with the Al sample dipped in the blank solution, the Al sample surface dipped in NaCl-containing inhibitor is relatively smooth, along with the absence of corrosion holes (Figure 8b). This is due to the formation of a protective film on the alloy surface. This film is constructed from the contribution of Ce, S, N, and C elements within the structure of inhibitor molecules (Table 3). This affirms the adsorption of inhibitor molecules on the alloy surface, which resists penetration and corrosion by aggressive Cl^−^ ions [65,66]. In particular, the large percentage of C, 7.8%, is due to the large molecular structure of complex molecules, which consist of many CH_2_ groups. As shown in Table 3, the amount of oxygen in this test sample diminishes substantially compared to the Al sample in the blank solution, indicating a reduction in the density of active sites where the oxidation reaction of AA2024-T3 alloy happens. This means a decrease in the quantity of Al_2_O_3_ oxide on the Al surface because of the presence of an inhibitor [67].

**Figure 9:**
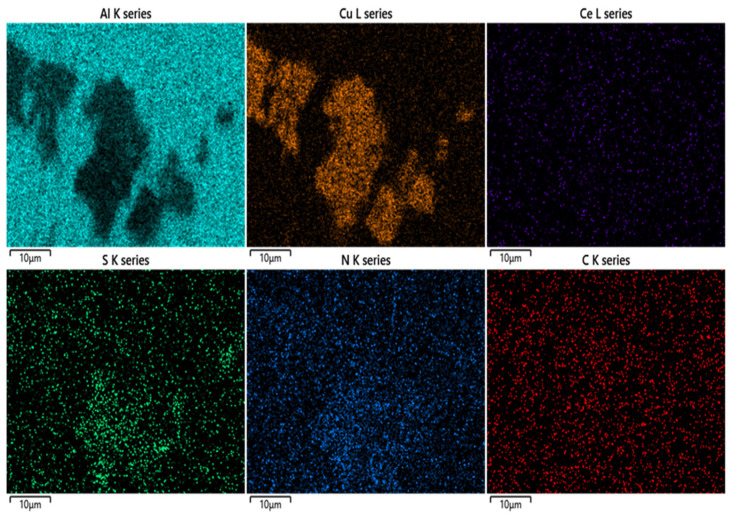
SEM-EDS mapping of the main element distribution in the AA 2024-T3 sample dipped in a 3.5% NaCl-containing inhibitor for 120 h.

Traces of precipitates are observed on the alloy surface dipped into a 3.5% NaCl solution containing an inhibitor (Figure 8b). Therefore, SEM-EDS mapping of this sample was performed to disclose the major composition as well as the reason for the formation of these precipitates. The results are displayed in Figure 9.

As observed in Figure 9, the precipitates are formed mainly from Cu elements originating from the Cu-rich particles of the S phase remnants after the de-alloying behaviour. However, these precipitates are formed from not only Cu-rich particles but also N and S elements. This suggests that the (TEPA/CS_2_^−^) organic parts of complex molecules were separated from Ce ions after this complex was dissolved in NaCl. Then, this organic part adsorbs and forms a film on the IMPs, which contain Cu-rich particles [68]. This film blocks the active sites and reduces the anodic dissolution of the alloy. Nonetheless, this layer is inadequate to completely cover the IMPs so there is a transition between the IMPs and the covered areas. This causes the flickering behaviour on the anodic branch in the potentiodynamic polarization test results (Figure 7b).

#### 3.3.2. XPS Analysis

**Figure 10:**
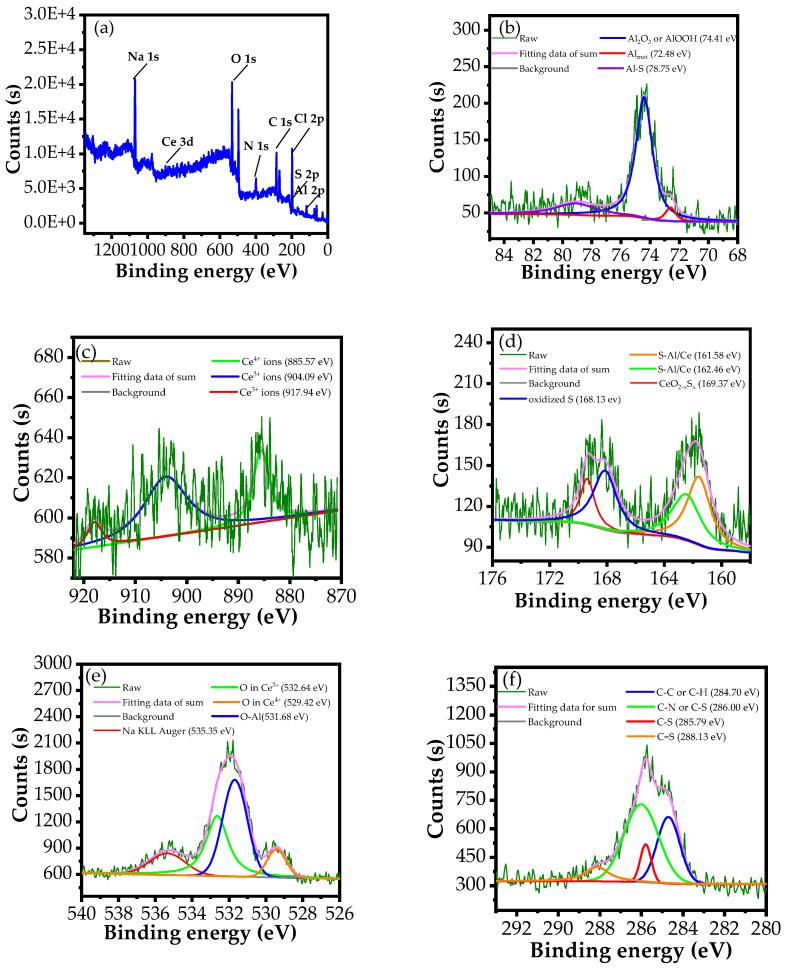
(**a**) XPS survey scan of AA 2024-T3 alloy surface immersed in a 3.5% NaCl-containing inhibitor. High-resolution scans of (**b**) Al 2p, (**c**) Ce 3d, (**d**) S 2p, (**e**) O 1s, and (**f**) C 1s.

The XPS technique was applied to investigate the chemical composition of the protective film formed on the metal surface in the NaCl solution in the presence of an inhibitor [69]. XPS spectra, as well as high-resolution spectra, of major components of the surface film formed on the AA 2024-T3 in the 3.5% NaCl-containing inhibitor over 120 h are displayed in Figure 10. Furthermore, the atomic percentage of these components is shown in Table 4.

As shown in Figure 10a and Table 4, the product film formed on AA 2024-T3 alloy in the 3.5% NaCl-containing inhibitor presents Al, Na, O, and Cl, along with C, N, S, and Ce elements, which are original components in the structure of the complex. This reveals the presence of inhibitor molecules in the surface product layer of AA2024-T3 alloy [1]. To obviously determine the formation mechanism of this surface product layer, high-resolution XPS spectra of these species were processed through a deconvolution fitting process. From Figure 10b, the Al signal shows three deconvoluted peaks at 74.41 eV, 72.48 eV, and 78.75 eV, which are assigned to Al_2_O_3_/AlOOH compounds, metallic Al, and Al in Al−S bonding, respectively [3,70]. From the high-resolution spectrum of Ce, there are three peaks labelled at binding energies of 885.57 eV, 904.09 eV, and 917.94 eV, which are associated with the valence states of Ce^4+^ and Ce^3+^, respectively (Figure 10c) [71]. The S spectrum is composed of four peaks, originating from S−Ce bonds located at 161.58 eV, S ions in low coordination state (S−Al) located at 162.46 eV [72,73], CeO_2−x_S_x_ compound positioned at 169.31 eV [74], and the oxidized S located at 168.13 eV [75] (Figure 10d). The O 1s spectrum shows three characteristic peaks at the binding energies of 532.64 eV, 529.42 eV, 531.68 eV, and 535.35 eV, related to the oxidation states of Ce^3+^ and Ce^4+^ [76,77], aluminium oxide compounds [78], and Na KLL Auger [79], respectively (Figure 10e). The XPS spectrum of C 1s appears four deconvolution peaks at 284.70 eV, 286.00 eV, 285.79 eV, and 288.13 eV, which are attributed to C−C/C−H [80], C−N/C−S [81], C−S [82], and C=S [83], respectively (Figure 10f). It is noteworthy in Table 4 that C and N species contribute significantly to the creation of surface product layers with 31.71 and 9.53%, respectively. This is derived from the large structure of inhibitor in the form of a hydrocarbon chain substituted by NH groups. This result is in accordance with the results obtained from SEM-EDS analysis.

According to the XPS results, the presence of inhibitor molecules on the alloy surface as well as the formation of new bonds between inhibitor molecules and the Al surface suggests that the inhibitor molecules absorb and generate a protective film on the Al alloy surface. This film isolates the alloy surface from the corrosive environment, leading to the improved corrosion resistance of the AA 2024-T3 alloy.

### 3.4. Corrosion Inhibition Mechanism

**Figure 11:**
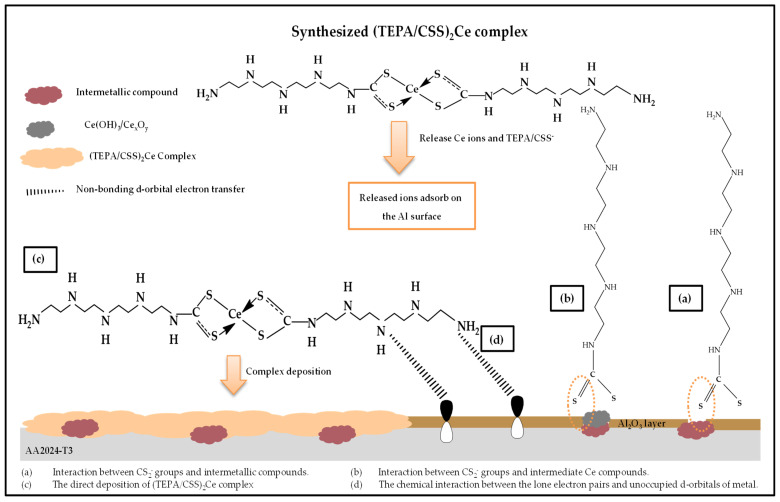
The corrosion protection mechanism of (TEPA/CSS)_2_Ce complex molecules on the AA 2024-T3 surface.

The adsorption mechanism of corrosion inhibitors containing organic parts on the metal surface is based on various aspects including the type of metal, the aggressive media, and characterizations of the inhibitor (functional groups presented in the molecular structure of organic parts) [84]. Furthermore, because of the naturally inhomogeneous surface of alloys, the surface introduces several adsorption sites where activation energies and heats of the adsorption process are divergent [85]. As a result, the adsorption mechanism of these corrosion inhibitors on the alloy surface cannot be clearly classified as either a chemical process or a physical process but is a combination of both processes. According to the results obtained from the electrochemical tests and surface analyses, the (TEPA/CSS)_2_Ce complex molecules are adsorbed on the AA2024-T3 alloy surface through the synergy of four mechanisms belonging to both chemisorption and physisorption (Figure 11).

(a) Interaction between CS_2_^−^ groups and intermetallic compounds.

Anti-corrosion efficiency is related to interactions between CS_2_^−^ groups and intermetallic compounds. When the (TEPA/CSS)_2_Ce complex is dissolved into NaCl solution, Ce and TEPA/CS_2_^−^ ions are released. After that, the CS_2_^−^ groups tend to interact with intermetallic compounds on the anodic sites of AA2024-T3 alloy through bridges between S atoms and metal ions (Figure 11a) [86]. This is proven through the presence of S−Al bonds in the XPS results. Furthermore, the deposition traces in the SEM images and flickering behaviour on the Tafel anodic branch also confirm this mechanism.

(b) Interaction between CS_2_^−^ groups and intermediate Ce compounds.

The simultaneous release of Ce and TEPA/CS_2_^−^ ions is responsible for the second mechanism. Firstly, released Ce ions deposit on the cathodic sites of AA 2024-T3 surface in the form of CeO_2_, Ce_2_O_3_, Ce(OH)_3,_ and Ce(OH)_4_ compounds [87]. Next, the CS_2_^−^ groups of TEPA/CS_2_^−^ parts bond to these oxide/hydroxide compounds (Figure 11b). The existence of the CeO_2-x_S_x_ phase, Ce^4+^, and Ce^3+^ states in the XPS analysis results verify that this mechanism is a part of the anti-corrosion effect.

(c) The direct deposition of the (TEPA/CSS)_2_Ce complex on the Al surface.

The direct deposition of complex molecules on the Al alloy surface leads to the formation of a film, which covers and protects the alloy surface against the strong attack of the NaCl corrodent (Figure 11c). This is identified by virtue of the presence of S−Ce, C−C/C−H, C−N/C−S, and C=S bonds in the XPS results.

(d) The chemical interactions between the lone electron pairs and unoccupied d-orbitals of the metal.

This mechanism comes from the structure of the complex molecules which contain a large number of N atoms. The lone-pair electrons on these N atoms interact chemically with the free d-orbitals of the Al metal, which favours the adsorption process of the complex on the AA2024-T3 alloy surface (Figure 11d).

Furthermore, the complex corrosion inhibitor is a long chain molecule that creates a higher Al surface coverage, leading to improved corrosion inhibition efficiency [25].

## 4. Comparison with Other Corrosion Inhibitors

The corrosion inhibition efficiency of (TEPA/CSS)_2_Ce complex on the AA2024-T3 alloy in a 3.5% NaCl solution for 120 h of immersion time is 96.8%, which is significantly higher than other corrosion inhibitors applied in the anti-corrosion for AA2024-T3 in 3.5% NaCl (Table 5).

## 5. Conclusions

In summary, we successfully synthesized cerium/tetraethylenepentamine dithiocarbamate complex and applied it to corrosion inhibition for AA 2024-T3 alloy in 3.5% NaCl. The obtained results of potentiodynamic polarization tests show that the complex plays a role as a mixed-type inhibitor with predominantly cathodic inhibitive effects and widens the passive state of the alloy. As a result, corrosion inhibition efficiency expresses an uptrend over the immersion time with 32.31% and 81.72% for 24 h and 120 h, respectively. EIS test results indicate that the polarization resistance of the Al alloy is increased when the complex is presented in a 3.5% NaCl solution. After 120 h of immersion time, the polarization resistances of Al in 3.5% NaCl without and with corrosion inhibitor are 8.79  × 10^3^ and 277.10 × 10^3^ Ω cm^2^, respectively. Moreover, the polarization resistance of the Al sample dipped in 3.5% NaCl in the presence of an inhibitor rises over the immersion time, leading to an increase in the corrosion inhibition efficiency with the immersion time, correspondingly. The results of SEM-EDS and XPS analyses confirm the formation of a protective film on the Al alloy surface when the inhibitor is added to the NaCl solution. This layer is derived from the adsorption of the inhibitor molecules on the Al surface through both chemical and physical processes. Moreover, the large structure of the complex also contributes to enhanced inhibition efficiency. The findings in this study open an avenue toward the synthesis and employment of large metal–dithiocarbamate complexes in corrosion inhibition for metals.

## Figures and Tables

**Table 1:** Electrochemical parameters extracted from EIS curves for AA2024-T3 in 3.5% NaCl in the absence and presence of the (TEPA/CSS)_2_Ce corrosion inhibitor over time.

Medium	Immersion Time (h)	R_s_ (Ω cm^2^)	Q_f_ (S cm^−2^s^n^)	n	R_f_ (Ω cm^2^)	C_dl_ (S cm^−2^s^n^)	R_ct_ (Ω cm^2^)	R_p_ (Ω cm^2^)	E (%)
3.5% NaCl	2	10.33	2.55 × 10^−5^	0.91	13.1 × 10^3^	0.24 × 10^−3^	8.22 × 10^3^	21.32 × 10^3^	-
	12	9.43	5.39 × 10^−5^	0.89	4.19 × 10^3^	1.66 × 10^−3^	5.94 × 10^3^	10.13 × 10^3^	-
	24	9.29	6.58 × 10^−5^	0.86	4.52 × 10^3^	2.34 × 10^−3^	6.36 × 10^3^	10.88 × 10^3^	-
	72	9.19	9.18 × 10^−5^	0.83	5.58 × 10^3^	2.05 × 10^−3^	9.00 × 10^3^	14.58 × 10^3^	-
	120	9.05	18.12 × 10^−5^	0.77	4.32 × 10^3^	6.18 × 10^−3^	4.47 × 10^3^	8.79 × 10^3^	-
3.5% NaCl + inhibitor	2	10.34	8.00 × 10^−5^	0.94	4.13 × 10^3^	2.16 × 10^−3^	4.85 × 10^3^	8.97 × 10^3^	-
	12	10.39	7.99 × 10^−5^	0.93	9.01 × 10^3^	1.48 × 10^−3^	7.37 × 10^3^	16.38 × 10^3^	33.55
	24	9.75	3.19 × 10^−5^	0.95	3.91 × 10^3^	1.39 × 10^−3^	14.2 × 10^3^	18.11 × 10^3^	39.91
	72	10.41	7.26 × 10^−5^	0.90	19.78 × 10^3^	0.66 × 10^−3^	24.03 × 10^3^	43.81 × 10^3^	66.72
	120	9.35	4.42 × 10^−5^	0.82	118.70 × 10^3^	0.12 × 10^−3^	158.40 × 10^3^	277.10 × 10^3^	96.80

**Table 2:** The potentiodynamic polarization parameters for AA 2024-T3 in a 3.5% NaCl-containing inhibitor (**a**) after 24 h of immersion time and (**b**) after 120 h of immersion time.

(a) 24 h					
**Solution**	***E_corr_*** **(V)**	***E_br_*** **(V)**	***E_pit_*** − ***E_corr_*** **(V)**	***I_corr_*** **(μA cm^−2^)**	***E*** **(%)**
3.5% NaCl	−0.63	-	-	489	-
3.5% NaCl + inhibitor	−0.77	−0.62	0.15	331	32.31
**(b) 120 h**					
**Solution**	***E_corr_*** **(V)**	***E_br_*** **(V)**	***E_pit_*** − ***E_corr_*** **(V)**	***I_corr_*** **(μA cm^−2^)**	***E*** **(%)**
3.5% NaCl	−0.76	−0.66	0.1	2390	-
3.5% NaCl + inhibitor	−0.89	−0.57	0.32	437	81.72

**Table 3:** The percentage of elements (atomic per cent) obtained from EDS spectra shown in Figure 8.

Solution	Al	O	Cu	Mg	Si	C	S	N	Ce
3.5% NaCl	71.1	21.6	5.3	0.9	1.1	-	-	-	-
3.5% NaCl + inhibitor	69.4	7.2	14.1	0.7	-	7.8	0.4	0.3	0.1

**Table 4:** Species present in the surface film of the test sample and their atomic percentages.

Elements	Cl 2p	Na 1s	O 1s	Al 2p	C 1s	N 1s	S 2p	Ce 3d
Atomic (%)	13.2	9.1	23.15	10.00	31.71	9.53	2.61	0.70

**Table 5:** Comparative corrosion inhibition efficiency of (TEPA/CSS)_2_Ce complex in this study with other corrosion inhibitors on AA2024-T3 in 3.5% NaCl.

Solutions	Immersion Time (hours)	Efficiency (%)	Year	References
3.5% NaCl + (E)-2-(2,4-dihydroxybenzylidene) Hydrazinecarbothioamide	120	90	2018	[1]
3.5% NaCl + (E)-2-(2-hydroxybenzylidence) hydrazinecarbothioamide	120	74.9	2018	[1]
3.5% NaCl + (TEPA/CSS)_2_Ce	120	96.8	2022	This study

## Data Availability

Not applicable.
